# Prescribing Patterns and Clinical Outcomes of Ceftolozane/Tazobactam by Renal Function and Body Mass Index: A SPECTRA Real-World Multi-Country Analysis

**DOI:** 10.3390/antibiotics15030303

**Published:** 2026-03-17

**Authors:** Emre Yucel, Alex Soriano, Florian Thalhammer, Stefan Kluge, Mike Allen, Jessica Levy, Huina Yang, Sunny Kaul

**Affiliations:** 1 Merck & Co., Inc., 126 East Lincoln Avenue, P.O. Box 2000, Rahway, NJ 07065, USA; 2 Department of Infectious Diseases, Hospital Clinic, Carrer de Villarroel, 170, L’Eixample, 08036 Barcelona, Spain; 3 Department of Urology, Medical University of Vienna, Spitalgasse 23, 1090 Vienna, Austria; 4 Department of Intensive Care Medicine, University Medical Center Hamburg-Eppendorf, Martini Street 52, 20251 Hamburg, Germany; 5 MSD (UK) Limited, 2 Pancras Sq, London N1C 4AG, UK; 6 Department of Laboratory Medicine, Tan Tock Seng Hospital, 11 Jln Tan Tock Seng, Singapore 308433, Singapore; 7 Royal Brompton & Harefield NHS Foundation Trust, Britten St, London SW3 6PY, UK

**Keywords:** ceftolozane/tazobactam, renal function, creatinine clearance, BMI, obesity, real-world, *Pseudomonas aeruginosa*, clinical outcomes, antimicrobial stewardship, SPECTRA

## Abstract

**Background:** Antimicrobial resistance is a global health crisis associated with high mortality and economic burden. Patients with renal dysfunction and obesity have increased susceptibility to infections and may experience different real-world outcomes, including clinical success and mortality, but are often under-represented in clinical trials. Ceftolozane/tazobactam (C/T) is an innovative therapy used to treat resistant Gram-negative infections. We aimed to describe real-world clinical outcomes in hospitalized adults treated with C/T across categories of renal function and BMI in the SPECTRA study. **Methods:** SPECTRA was a multi-national observational study on 617 patients who received C/T for ≥48 h. Outcomes included clinical success, all-cause in-hospital mortality, readmission, and ICU admission and length of stay (LOS), with sub-analysis of patients across BMI and renal function strata. **Results:** Renal function and weight were reported in 597 and 469 patients, respectively, of which 51.9% had lower creatine clearance (<80 mL/min) and 50.7% were overweight. Clinical success and all-cause in-hospital mortality ranged at 59.1–77.8% and 11.1–29.2% across renal function strata and 64.6–68.6% and 18.6–21.4% across weight subgroups. Across renal function and weight subgroups, 38.9–54.2% and 45.9–53.5% of patients were admitted to ICU. Median ICU LOS was 8–21.5 and 14–20 days, respectively. Readmission (30-day all-cause) occurred in 4.5–11.8% and 8.2–11.9% of patients across renal function and weight strata. **Conclusions:** Results from this sub-analysis suggest real-world clinical effectiveness of C/T across patients with renal impairment and obesity, highlighting C/T as a component within treatment guidelines for resistant Gram-negative infections.

## 1. Introduction

Antimicrobial resistance (AMR) is a global health crisis. If current trends continue, AMR is projected to cause an estimated 10 million deaths per year worldwide and a global economic burden of $100 trillion by 2050 [1]. The rise of multidrug-resistant (MDR) organisms, particularly Gram-negative bacteria such as *Klebsiella pneumoniae*, *Acinetobacter baumannii*, *Pseudomonas aeruginosa* (PsA), and Enterobacter species have rendered many traditional antibiotics ineffective [2,3,4]. Despite this, the development of new antibiotics has slowed, with only a limited number of novel agents approved in recent years [5]. Innovative therapies such as ceftolozane/tazobactam (C/T) have emerged to address this gap, offering potent activity against resistant Gram-negative bacteria including PsA [6].

Renal dysfunction and obesity are associated with altered host physiology and immune responses and are linked to a higher risk of infection and worse infection-related outcomes in observational studies [7,8,9]. Approximately 700 million people are affected by chronic kidney disease globally [10]. Patients with severe renal impairment experience among the highest rates of antimicrobial-resistant infections, which are a leading cause of mortality in those with end-stage renal disease [11]. Renal dysfunction (creatinine clearance [CrCL] ≤ 80 mL/min) markedly alters antibiotic pharmacokinetics—including clearance, volume of distribution, and half-life—necessitating dose adjustments as well as influencing monitoring strategies and therapeutic selection to minimize nephrotoxicity and avoid subtherapeutic exposure [12]. The burden of obesity is also significant, affecting more than 890 million adults globally [13]. Obesity is associated with a number of complications, including reduced wound healing and respiratory compromise that can change treatment decisions and outcomes [8]. Obesity is also associated with increased in-hospital mortality among patients receiving treatment for infections [14]. Additionally, obesity can increase volume of distribution and, depending on clearance, result in lower plasma concentrations adding complexity to the management of infectious diseases [15,16]. Renal impairment and obesity often co-exist, compounding risks and complicating treatment strategies by independently and interactively influencing antimicrobial pharmacokinetics/pharmacodynamics (PK/PD) [17]. Documenting C/T treatment and outcomes in patients with renal impairment and obesity helps to provide a full picture of real-world prescribing patterns.

While randomized controlled trials (RCTs) remain the gold standard, they often exclude patients with comorbidities such as renal dysfunction or obesity [18]. Real-world evidence complements RCTs by evaluating treatment effectiveness in routine clinical settings, capturing diverse populations and long-term outcomes [19], and is increasingly used to inform regulatory decisions, clinical guidelines, and health technology assessments [20]. Describing current real-world practice helps to identify gaps between guideline dosing advice and clinical practice.

The Study of Prescribing patterns and Effectiveness of Ceftolozane/Tazobactam Real-world Analysis (SPECTRA) provides a large, multi-country dataset with detailed clinical and microbiology data. Previous publications have disseminated data, summarizing its overall findings and findings in critically ill as well as in pulmonary disease- and respiratory-related infection cohorts [21,22,23]. This manuscript combined renal function and obesity (according to body mass index [BMI]) sub-analyses with the objective of describing clinical success, all-cause in-hospital mortality, intensive care unit (ICU) admission and length, and readmission in adults treated with C/T for ≥48 h while hospitalized. In this analysis, renal impairment is defined by creatinine clearance categories and includes patients with diverse underlying causes (e.g., diabetes, hypertension, other chronic kidney disease, and acute kidney injury).

## 2. Results

### 2.1. Patient Characteristics

#### 2.1.1. Renal Function

Renal function data were reported for 597 of the 617 patients included in the SPECTRA study, while 20 patients (3.2%) had missing renal function data. Of those with known renal data, 51.9% were categorized as having LCC. Mild renal impairment was most reported (25.6%), followed by moderate renal impairment (21.3%; Table 1). Mean age was lowest in patients with augmented renal impairment (42.8 years) and normal renal function (51.4 years), and it increased in patients with any severity of renal impairment or LCC (59.2–63.1 years; Table 1). Mean BMI was similar across renal function categories, with slight peaks for the augmented renal function (28.2 kg/m^2^) and severe renal impairment groups (27.0 kg/m^2^; Table 1).

#### 2.1.2. BMI

BMI was recorded in 469 of the 617 patients enrolled in the study, while 148 patients (24.0%) had missing BMI data. Of those patients with known BMI, 194 (41.4%) were considered normal weight, 159 (33.9%) were overweight, and 79 (16.8%) were obese. Mean age was higher in patients who were overweight (60.3 years) or obese (61.8 years) than those with normal weight (52.7 years; Table 2).

### 2.2. Clinical Outcomes

#### 2.2.1. Renal Function

Clinical success was observed in 70.3% (95% CI: 63.9, 76.1) of patients with normal renal function and 64.8% (95% CI: 59.2, 70.2) patients with LCC (Table 3). In patients with LCC, observed clinical success was similar regardless of the level of renal impairment (mild: 73.9% [95% CI: 66.1, 80.6]; moderate: 59.1% [95% CI: 50.0, 67.7]; severe: 60.4% [95% CI: 45.3, 74.2]; end-stage: 63.6% [95% CI: 40.7, 82.8]) (Table 3).

During the index hospitalization, 48.5% of patients with normal renal function were admitted to the ICU, of which 37.8% of ICU admissions were related to the index infection. The median ICU length of stay (LOS) was 19 days. Similarly, 58.1% of patients with LCC were admitted to the ICU, of which 43.6% of ICU admissions were related to the index infection. Median ICU LOS in patients with LCC was 15.5 days. When considering level of renal impairment, ICU admission rates were 42.5% for patients with mild renal impairment, 49.6% in patients with moderate renal impairment, 54.2% in patients with severe renal impairment, and 50.0% in patients with end-stage renal impairment. Of those, 43.1%, 41.3%, 53.8%, and 36.4% were related to the index infection, respectively. Median ICU LOS ranged from 10 to 21.5 days, with the highest LOS observed in patients with severe renal impairment (Table 3). Regardless of renal function, 30-day all-cause readmission was generally low (4.5–12.5%; Table 3).

All-cause in-hospital mortality was 17.0% (95% CI: 12.4, 22.5) for patients with normal renal function and 22.3% (95% CI: 12.4., 22.5) for patients with LCC (Table 3). Across severities of renal impairment, all-cause in-hospital mortality was 17.6% (95% CI: 12.0, 24.6) in patients with mild renal impairment, 26.8% (95% CI: 19.3, 35.4) in patients with moderate renal impairment, 29.2% (95% CI: 17.0, 44.1) in patients with severe renal impairment, and 27.3% (95% CI: 10.7, 50.2) in patients with end-stage renal impairment (Table 3).

#### 2.2.2. BMI

Observed clinical-success rates were similar across BMI categories, reported in 68.6% (95% CI: 61.5, 75.0) of patients who were classed as normal weight, 67.9% (95% CI: 60.1, 75.1) of patients who were overweight, and 64.6% (95% CI: 53.0, 75.0) of patients who were obese (Table 4).

During the index hospitalization, 49.9% of patients who were normal weight, 53.5% of patients who were overweight, and 50.6% of patients who were obese were admitted to the ICU. Of these ICU admissions, 47.2%, 29.4%, and 47.5% were related to the index infection, respectively. The median ICU LOS increased with BMI at 14, 19, and 20 days, respectively. Thirty-day all-cause and infection-related readmissions were generally low regardless of BMI (8.8–11.9% and 2.5–6.3%, respectively), with roughly half of all readmissions related to the index infection (Table 4).

Observed all-cause in-hospital mortality rates were similar across BMI categories, occurring in 18.6% (CI: 13.3, 24.8) of patients who were normal weight, 21.4% (CI: 15.3, 28.6) of patients who were overweight, and 19.0% (CI: 11.0, 29.4) of patients who were obese (Table 4).

## 3. Discussion

In the SPECTRA study, C/T was administered to patients across the spectrum of renal function and BMI categories. Observed clinical success rates were similar across renal function (59.1% [95% CI: 50.0, 67.7]—77.8% [95% CI: 52.4, 93.6) and BMI categories (64.6% [95% CI: 53.0, 75.0]—68.6% [95% CI: 61.5, 75.0]). These findings are similar to those reported in clinical trials, which found that patients with mild and moderate renal impairment had a response rate of 48–81%, depending on the infection and renal impairment severity [24]. These findings suggest that C/T may remain an appropriate and effective treatment for use in patients across renal function and BMI categories. However, no adjusted or formal equivalence/non-inferiority testing was performed.

Observed all-cause in-hospital mortality following C/T treatment was similar across BMI categories (18.6% [95% CI: 13.3, 24.8]—21.4% [95% CI: 15.3, 28.6]), suggesting that C/T efficacy may not be influenced by BMI. However, no adjusted or formal equivalence/non-inferiority testing was performed to confirm this finding. Differently, observed all-cause in-hospital mortality was higher among patients with moderate to end-stage renal impairment (26.8% [95% CI: 19.3, 35.4]—29.2% [95% CI: 17.0, 44.1]) than among those with augmented, normal, or mild renal function (11.1% [95% CI: 1.4, 34.7]—17.6% [95% CI: 12.0, 24.6]). However, these results are unadjusted and may reflect baseline risk, differences in care, or other confounding factors rather than an independent effect of renal function. The proportional increase in all-cause in-hospital mortality seen within the renal cohort in this study may also suggest a difference in PK/PD based on renal function [25]. This aligns with a previous study which identified an association between drug molecular weight and PK alternations in patients with renal impairment. As a result, Food and Drug Administration (FDA) guidelines’ recommend dose of C/T is adjusted based on CrCl [26,27].

Observed ICU admissions, both all-cause and related to the index infection, were generally similar across the spectrum of renal function (38.9–52.4% and 36.1–43.6%, respectively) and BMI categories (45.9–43.5% and 29.4–47.5%, respectively). However, no adjusted or formal equivalence/non-inferiority testing was performed. Observed mean ICU LOS was similar across renal function categories (21.8–24.5 days), except in patients with augmented renal function where mean ICU LOS was shorter (13.0 days). For BMI categories, ICU LOS slightly increased from normal weight (22.0 days) to overweight (23.3 days) and obese categories (25.1), but the difference is minimal and could likely be due to comorbidities that are common in obesity [28].

Across renal function and BMI categories, 30-day all-cause readmission rates remained consistent, occurring in 4.5–11.8% and 8.2–11.9% of patients, respectively. Index infection-related readmissions were also consistent across renal function and BMI categories, occurring in 0–8.3% and 2.5–6.3% of patients, respectively. These findings suggest that observed renal impairment and high BMI were not associated with higher levels of readmissions, suggesting the effectiveness of C/T in these populations may be comparable to patients with normal renal function and weight, although no adjusted or formal equivalence/non-inferiority testing was performed.

According to this study, observed clinical success, mortality, ICU admissions and LOS, and readmissions were similar across BMI categories, with minimal differences observed in patients who were obese. Literature on the effect of obesity on PK/PD is limited and often conflicting, and there currently are no guidelines in place to recommend C/T dose adjustments according to BMI [16,27]. However, dose adjustments for patients with obesity may be considered on a case-by-case basis [16]. As a result, the C/T dose administered to patients with obesity in this study may have been adjusted to account for weight differences that may have contributed to the observed consistency in clinical success and mortality. However, since this study did not collect individual dosing information, this cannot be confirmed [16].

The results of this study support the use of C/T in patients with AMR infections, regardless of renal function or BMI. Dose adjustment may be required in patients according to renal function, among other considerations. While we cannot confirm individual dosing or exposure, the observed overall outcomes are consistent with real-world use of label-based dosing in routine care; however, we cannot determine whether clinicians adjusted dosing in individual cases. Recommendations for future research include the evaluation of clinical outcomes by dose adjustments and serum levels in renally impaired patients and further analysis of the effectiveness of C/T in patients who have renal impairment and obesity in conjunction.

## 4. Materials and Methods

SPECTRA was a multi-national, multicenter, retrospective study of adult inpatients treated with C/T for ≥48 h, designed to provide real-world data on outcomes associated with C/T use. Data were extracted from medical records from January 2016 to November 2020 across seven countries. The study design, population and core outcome definitions are reported elsewhere [21,22,23]. Patients were included if aged ≥18 years, had received C/T for ≥48 h and had received their last C/T dose ≥30 days before data collection. Patients were excluded if they were participating in an interventional clinical trial for Gram-negative infection at the time of treatment.

The primary objective was to characterize real-world treatment patterns, clinical outcomes, and healthcare resource utilization associated with C/T in hospitalized adults who received ≥48 h of therapy. A key secondary objective was to analyze outcomes according to categories of renal function and obesity (categorized according to BMI), as reported in this manuscript.

For the purpose of this exploratory, descriptive analysis of patients treated with C/T as per the label, renal function was categorized by creatinine clearance (CrCl) using the SAP-defined thresholds: end-stage < 15 mL/min, severe 15–29 mL/min, moderate 30–59 mL/min, mild 60–89 mL/min, normal 90–149 mL/min, and augmented renal function ≥ 150 mL/min. These definitions of augmented renal function are used consistently throughout the analyses. BMI categories followed World Health Organization (WHO, Geneva, Switzerland) conventions: normal weight 18.5–<25 kg/m^2^; overweight 25–<30 kg/m^2^; obese ≥30 kg/m^2^. Within the analyses, percentages exclude patients with missing CrCl or BMI data as per the SAP.

CrCl was estimated using the Cockcroft–Gault formula from the most recent pre-treatment serum creatinine and patient demographic data in the chart, in accordance with the SAP. For patients who subsequently received renal replacement therapy (RRT) during the index hospitalization, CrCl was assigned based on the most recent available serum creatinine measured prior to initiation of RRT when that value was available and interpretable. If no pre-RRT creatinine value suitable for Cockcroft–Gault estimation was available, or if the record indicated ongoing RRT such that a steady-state CrCl could not be reasonably estimated, CrCl was considered missing and the patient was excluded from CrCl-stratified tables (consistent with the SAP available-case rules).

Table denominators use available-data rules as set out in the SAP; differences between table category sums and the full study population (N = 617) are due to exclusion of missing or non-derivable values and to the SAP cut points used for category assignment.

C/T was administered according to approved labeling and protocolized dosing specified in the study (Table S2). As dosing was applied per these label-based SAP recommendations, individual administered dose records were not collected during this analysis. No inferential statistical comparisons (e.g., adjusted regression) were performed. In addition, individual per-patient administered dose records, timing, serum concentrations, and MIC distributions were not collected for this analysis and therefore PK/PD or exposure–response assessments could not be performed.

Thirty-day readmissions were captured only within the index hospital’s medical records; readmissions at other hospitals or via national registries were not collected, and no additional follow-up to ascertain out-of-hospital readmissions was performed.

The study was conducted in compliance with the International Society for Pharmacoepidemiology Guidelines for Good Pharmacoepidemiology Practices [29], the ethical principles arising from the Declaration of Helsinki [30], the European Union good pharmacovigilance practices [31], European and national laws with respect to data protection [32], and applicable local regulations. In each participating country, the study was submitted to the relevant ethics committee and/or institutional review board as required, and approval or waiver of informed consent was obtained in accordance with local regulations; documentation of these approvals and waivers is held by the sponsor and participating sites.

## 5. Outcomes

Data were retrospectively collected from medical records covering up to 30 days after treatment or until death. Outcomes assessed included investigator-assessed clinical success of C/T treatment during index infection, all-cause in-hospital mortality, ICU admissions, ICU length of stay, and readmission rates. For the renal function cohort, information on renal replacement therapy and acute kidney injury can be found in the Supplementary Materials (Table S1).

Clinical success was defined as microbiological eradication, no Gram-negative therapy needed for a minimum of 48 h after administration of C/T (not including discharge antibiotics or de-escalation), no death due to Gram-negative infection, no additional treatment for exacerbation of respiratory infection within ≤28 days of stopping C/T, resolution of any exacerbations, or no need for re-operation for source control. Microbiology data (including repeat cultures and MIC distributions) were incompletely captured and therefore not analyzed by renal/BMI strata in this report. Outcomes reporting on the full included patient population are reported elsewhere [21].

## 6. Statistical Analysis

Categorical variables were reported as counts and percentages and continuous data were reported as mean and standard deviation (SD) or median and interquartile range (IQR). Where available, 95% confidence intervals (CIs) for proportions (mortality, clinical success) have been reported. No adjusted or formal equivalence/non-inferiority testing was performed.

Mean, SD, and median were not reported for CrCl due to some sites reporting all patients with normal renal function as 90 mL/min. Analysis of CrCl was thus categorized. Cross-tabulations were used to describe overlap between BMI and renal function categories. A response of ‘Unknown’ was handled as missing data and not included in the analysis. No inferential statistical comparisons (e.g., adjusted regression) were performed.

## 7. Limitations

The findings presented within this manuscript are and hypothesis-generating and descriptive in nature. Adjusted inference would require prospectively designed or specifically resourced analyses that were not included within the scope of this research.

The principal limitation of this analysis is the absence of individual-level administered dose and timing records. C/T was administered according to approved labeling and the study dosing table (Table S2), which defines recommended doses by indication and CrCl. However, patients with missing CrCl recordings or unknown infection type did not have defined doses (Table S2). Because individual administered dose and exposure data (including exact dose and timing, drug concentrations, and MIC distributions) were not collected as part of this descriptive, label-based analysis, exposure–response and PK/PD analyses were not possible. Consequently, the observed, unadjusted outcome rates reported here cannot be used to infer causal equivalence of C/T effectiveness across renal function or BMI strata; prospective studies or pooled datasets with detailed exposure and susceptibility data would be required for such inferences. Observed differences in clinical success or mortality across renal function and BMI strata may therefore reflect unmeasured factors, such as baseline severity of illness, clinician selection, site-level practice differences, or other confounders rather than effects of dosing.

Missing data, particularly for BMI (24.0%), is a limitation of this retrospective, multi-national dataset and may bias descriptive comparisons by BMI category. Although multiple imputation can be appropriate when data are plausibly missing at random and sufficient auxiliary predictors are consistently available, the heterogeneous patterns of missingness and limited auxiliary data across centers made MI inappropriate for the primary descriptive analysis; imputations under these conditions could introduce model-driven bias. We therefore present available-case descriptive results, provide transparent counts of missingness, and recommend that future prospectively designed studies or pooled datasets with more complete auxiliary variables be used for imputation-based sensitivity or adjusted inference.

Safety data were not collected as part of the SPECTRA study and safety assessments are beyond the remit of this study. Additional limitations include the retrospective design, potential selection bias, and heterogeneity in microbiology sampling and susceptibility testing across centers.

Despite these constraints, the findings of this study offer descriptive, real-world insight into C/T use and outcomes among patients with different renal function and BMI categories.

## 8. Conclusions

In this descriptive, retrospective SPECTRA, C/T was associated with clinical success across a range of renal function and BMI categories which may support the inclusion of C/T in treatment guidelines as a component in the treatment of patients with MDR infections, irrespective of renal function or BMI. However, given the study’s retrospective design, missing per-patient dosing data, and absence of adjusted analyses, these findings are hypothesis-generating and should not be interpreted as evidence of equivalence across subgroups. Nevertheless, this real-world evidence from diverse healthcare settings may help to support the broader adoption of C/T in clinical practice. Further research is warranted to better understand dosing in renally impaired patients for which the descriptive data from the SPECTRA study provides a basis for continuing such work.

## Figures and Tables

**Table 1:** Patient characteristics by renal function *.

	Augmented Renal Function	Normal Renal Function	Mild Renal Impairment	Moderate Renal Impairment	Severe Renal Impairment	End-Stage Renal Impairment	Total Study Population
	N = 18	N = 229	N = 153	N = 127	N = 48	N = 22	N = 617 ^†^
**Age (years) ^‡^**							
Mean (SD)	42.8 (16.6)	51.4 (17.2)	63.0 (15.7)	61.8 (16.1)	63.1 (15.1)	59.2 (14.6)	57.4 (17.3)
Median	39	54	64	64	65	59	59
Q1; Q3	31.0; 57.0	37.5; 65.0	51.5; 75.0	50.0; 76.0	52.0; 74.0	54.0; 68.0	45.0; 71.0
**Male (n [%])**	10 (55.6)	146 (63.8)	104 (68.0)	91 (71.7)	25 (52.1)	15 (68.2)	404 (65.5)
**BMI (kg/m^2^)**							
Mean (SD)	28.2 (7.2)	25.1 (7.6)	25.7 (6.5)	26.0 (5.0)	27.0 (6.4)	24.1 (6.4)	25.6 (6.7)
Median	27	24.7	24.9	26.2	25.3	23.4	25
Q1; Q3	23.1; 30.9	21.1; 27.0	21.5; 28.0	22.8; 29.1	22.3; 31.4	19.4; 26.7	21.6; 28.2

* Creatinine clearance (CrCl) was derived and categorized per the Statistical Analysis Plan. Category cut points used for the table are as follows: end-stage < 15 mL/min; severe 15–29 mL/min; moderate 30–59 mL/min; mild 60–89 mL/min; normal 90–149 mL/min; augmented ≥ 150 mL/min. Percentages in this table use the number of patients with non-missing CrCl as the denominator (CrCl recorded in 597/617 patients). ^†^ The cohort size of the total study population was 617. Renal function was not recorded for 20 patients; percentages in this table use the 597 patients with non-missing CrCl as the denominator. Seventy-one patients had RRT initiated during the index hospitalization (Table S1). When a pre-RRT serum creatinine suitable for Cockcroft–Gault estimation was available, the pre-RRT CrCl was used to assign renal function category. Patients without an interpretable pre-RRT creatinine were categorized as missing CrCl and therefore excluded from CrCl-stratified denominators (CrCl recorded for 597/617 patients). ^‡^ For patients aged 90 or older, the HCP was asked not to enter the exact age and to check a specific box. Therefore, patients aged 90 or older are included in ‘Missing’ and are not taken into account in the calculation of mean, median, etc. BMI: Body mass index; LLC: Lower Creatine Clearance; Q: Quartile; SD: Standard Deviation.

**Table 2:** Patient characteristics by BMI category.

	Normal Weight	Overweight	Obese	Total Study Population
	N = 194	N = 159	N = 79	N = 617 *
**Age (years) ^†^**				
Mean (SD)	52.7 (17.1)	60.3 (15.4)	61.8 (15.3)	57.4 (17.3)
Median	55	63	63	59
Q1; Q3	40.0; 65.0	51.0; 71.0	53.0; 74.0	45.0; 71.0
**Male (n [%])**	132 (68.0)	112 (70.4)	46 (58.2)	404 (65.5)
**BMI (kg/m^2^)**				
Mean (SD)	22.1 (1.9)	27.0 (1.5)	36.0 (8.2)	25.6 (6.7)
Median	22.1	26.9	33.3	25
Q1; Q3	20.5; 23.7	25.8; 28.2	31.5; 37.6	21.6; 28.2

* Patients with missing information on BMI are not presented in the table. For that reason, the sum of Ns in the BMI columns is inferior to the total N. BMI was derivable for 469/617 patients; percentages use the 469 patients with non-missing BMI as the denominator. The remaining patients either had missing BMI data (148), or records were flagged as implausible during data cleaning and so were excluded (n = 37). ^†^ For patients aged 90 or older the HCP was asked not to enter the exact age and to check a specific box. Therefore, patients aged 90 or older are included in ‘Missing’ and are not taken into account in the calculation of mean, median, etc. BMI: Body mass index; Q: Quartile; SD: Standard Deviation.

**Table 3:** Clinical outcomes by renal function *.

	Augmented Renal Function	Normal Renal Function	Mild Renal Impairment	Moderate Renal Impairment	Severe Renal Impairment	End-Stage Renal Impairment	Total Study Population
	N = 18	N = 229	N = 153	N = 127	N = 48	N = 22	N = 617 ^†^
**Index infection considered as a clinical success by the investigator (n [%])**	14 (77.8)	161 (70.3)	113 (73.9)	75 (59.1)	29 (60.4)	14 (63.6)	415 (67.3)
95% CI	(52.4, 93.6)	(63.9, 76.1)	(66.1, 80.6)	(50.0, 67.7)	(45.3, 74.2)	(40.7, 82.8)	(63.4, 71.0)
**All-cause in-hospital mortality (n [%])**	2 (11.1)	39 (17.0)	27 (17.6)	34 (26.8)	14 (29.2)	6 (27.3)	131 (21.2)
95% CI	(1.4, 34.7)	(12.4, 22.5)	(12.0, 24.6)	(19.3, 35.4)	(17.0, 44.1)	(10.7, 50.2)	(18.1, 24.7)
**Admission to ICU during the index hospitalization (n [%])**	7 (38.9)	111 (48.5)	65 (42.5)	63 (49.6)	26 (54.2)	11 (50.0)	298 (48.3)
**ICU related to index infection (n [%])**	3 (42.9)	42 (37.8)	28 (43.1)	26 (41.3)	14 (53.8)	4 (36.4)	125 (41.9)
**Median ICU length of stay (days)**	8	19	21	10	21.5	16.5	19
Q1; Q3	6.0; 25.0	8.0; 37.0	9.0; 38.5	5.0; 37.0	8.0; 45.0	4.0; 39.0	8.0; 37.5
30-day all-causes readmission ** (n [%])	1 (5.6)	22 (9.6)	18 (11.8)	13 (10.2)	6 (12.5)	1 (4.5)	63 (10.2)
30-day infection-related readmission ** (n [%])	0	9 (3.9)	9 (5.9)	6 (4.7)	4 (8.3)	0	30 (4.9)

* Creatinine clearance (CrCl) was derived and categorized per the Statistical Analysis Plan. Category cut points used for the table are as follows: end-stage < 15 mL/min; severe 15–29 mL/min; moderate 30–59 mL/min; mild 60–89 mL/min; normal 90–149 mL/min; augmented ≥ 150 mL/min. Percentages in this table use the number of patients with non-missing CrCl as the denominator (CrCl recorded in 597/617 patients). ^†^ The cohort size of the total study population was 617. Renal function was not recorded for 20 patients; percentages in this table use the 597 patients with non-missing CrCl as the denominator. Dosing followed SAP guidance by indication and CrCl (Table S2); individual administered doses were not collected. ** Thirty-day readmissions were captured only within the index hospital’s medical records; readmissions at other hospitals or via national registries were not collected, and no additional follow-up to ascertain out-of-hospital readmissions was performed. CI: Confidence interval; ICU: Intensive Care Unit; LLC: Lower Creatine Clearance; SD: Standard Deviation.

**Table 4:** Clinical outcomes by BMI category.

	Normal Weight	Overweight	Obese	Total Study Population
	N = 194	N = 159	N = 79	N = 617 *
**Index infection considered as a clinical success by the investigator (n [%])**	133 (68.6)	108 (67.9)	51 (64.6)	415 (67.3)
95% CI	(61.5, 75.0)	(60.1, 75.1)	(53.0, 75.0)	(63.4, 71.0)
**All-cause in-hospital Mortality (n [%])**	36 (18.6)	34 (21.4)	15 (19.0)	131 (21.2)
95% CI	(13.3, 24.8)	(15.3, 28.6)	(11.0, 29.4)	(18.1, 24.7)
**Admission to ICU during the index hospitalization (n [%])**	89 (45.9)	85 (53.5)	40 (50.6)	298 (48.3)
**ICU related to index infection (n [%])**	42 (47.2)	25 (29.4)	19 (47.5)	125 (41.9)
**Median ICU length of stay (days)**	14	19	20	19
Q1; Q3	7.0; 32.0	8.0; 34.0	8.0; 41.0	8.0; 37.5
30-day all-causes readmission ** (n [%])	23 (11.9)	13 (8.2)	8 (10.1)	63 (10.2)
30-day infection-related readmission ** (n [%])	10 (5.2)	4 (2.5)	5 (6.3)	30 (4.9)

* Patients with missing information on BMI are not presented in the table. For that reason, the sum of Ns in the BMI columns is inferior to the total N. BMI was derivable for 469/617 patients; percentages use the 469 patients with non-missing BMI as the denominator. The remaining patients either had missing BMI data (148) or records were flagged as implausible during data cleaning and were thus excluded (n = 37). ** Thirty-day readmissions were captured only within the index hospital’s medical records; readmissions at other hospitals or via national registries were not collected, and no additional follow-up to ascertain out-of-hospital readmissions was performed. CI: Confidence interval; ICU: Intensive Care Unit; SD: Standard Deviation.

## Data Availability

The full datasets generated and analyzed to inform the conclusions drawn within this manuscript during the current study are available from the corresponding author upon reasonable request.

## Supplementary Materials

The following supporting information can be downloaded at https://www.mdpi.com/article/10.3390/antibiotics15030303/s1. Table S1. Renal replacement therapy types and duration*. Table S2. Appropriate Dose of C/T for Index Event.

## References

[1] Berger I., Loewy Z.G. (2024). Antimicrobial resistance and novel alternative approaches to conventional antibiotics. Bacteria.

[2] Centers for Disease Control Prevention Management of Multidrug-Resistant Organisms in Healthcare Settings. https://www.cdc.gov/infection-control/hcp/mdro-management/background.html.

[3] World Health Organization (2024). Antimicrobial Resistance. https://www.who.int/news-room/fact-sheets/detail/antimicrobial-resistance.

[4] World Health Organization (2024). WHO Updates List of Drug-Resistant Bacteria Most Threatening to Human Health.

[5] World Health Organization (2025). WHO Releases New Reports on New Tests and Treatments in Development for Bacterial Infections. https://www.who.int/news/item/02-10-2025-who-releases-new-reports-on-new-tests-and-treatments-in-development-for-bacterial-infections.

[6] Losito A.R., Raffaelli F., Del Giacomo P., Tumbarello M. (2022). New Drugs for the Treatment of *Pseudomonas aeruginosa* Infections with Limited Treatment Options: A Narrative Review. Antibiotics.

[7] León-Román J., Azancot M.A., Marouco C., Patricio-Liebana M., Zamora J.I., Terrades N.R., Toapanta N., Núñez-Delgado S., Fernandez A.B.M., Soler M.J. (2025). A New Era in the Management of Cardiorenal Syndrome: The Importance of Cardiorenal Units. Cardiorenal Med..

[8] Pugliese G., Liccardi A., Graziadio C., Barrea L., Muscogiuri G., Colao A. (2022). Obesity and infectious diseases: Pathophysiology and epidemiology of a double pandemic condition. Int. J. Obes..

[9] Ray A., Bonorden M.J.L., Pandit R., Nkhata K.J., Bishayee A. (2023). Infections and immunity: Associations with obesity and related metabolic disorders. J. Pathol. Transl. Med..

[10] Francis A., Harhay M.N., Ong A.C.M., Tummalapalli S.L., Ortiz A., Fogo A.B., Fliser D., Roy-Chaudhury P., Fontana M., Nangaku M. (2024). Chronic kidney disease and the global public health agenda: An international consensus. Nat. Rev. Nephrol..

[11] Wang T.Z., Kodiyanplakkal R.P.L., Calfee D.P. (2019). Antimicrobial resistance in nephrology. Nat. Rev. Nephrol..

[12] Corona A., Cattaneo D., Latronico N. (2022). Antibiotic Therapy in the Critically Ill with Acute Renal Failure and Renal Replacement Therapy: A Narrative Review. Antibiotics.

[13] World Health Organisation (2025). Obesity and Overweight. https://www.who.int/news-room/fact-sheets/detail/obesity-and-overweight.

[14] Narayanan N., Lin T., Vinarov D., Bucek T., Johnson L., Mathew C., Chaudhry S., Brunetti L. (2021). Relationship Between Multidrug-Resistant Enterobacterales and Obesity in Older Adults. Infect. Drug Resist..

[15] Bakdach D., Elajez R., Bakdach A.R., Awaisu A., De Pascale G., Ait Hssain A. (2022). Pharmacokinetics, Pharmacodynamics, and Dosing Considerations of Novel β-Lactams and β-Lactam/β-Lactamase Inhibitors in Critically Ill Adult Patients: Focus on Obesity, Augmented Renal Clearance, Renal Replacement Therapies, and Extracorporeal Membrane Oxygenation. J. Clin. Med..

[16] Castro-Balado A., Varela-Rey I., Mejuto B., Mondelo-García C., Zarra-Ferro I., Rodríguez-Jato T., Fernández-Ferreiro A. (2024). Updated antimicrobial dosing recommendations for obese patients. Antimicrob. Agents Chemother..

[17] Yim H.E., Yoo K.H. (2021). Obesity and chronic kidney disease: Prevalence, mechanism, and management. Clin. Exp. Pediatr..

[18] Kennedy-Martin T., Curtis S., Faries D., Robinson S., Johnston J. (2015). A literature review on the representativeness of randomized controlled trial samples and implications for the external validity of trial results. Trials.

[19] Devane D., Emir B., Watt S., O’Donnell M. (2025). Beyond the binary: Integrating “real-world evidence” with randomized trials in contemporary health care. J. Clin. Epidemiol..

[20] Candore G., Martin C., Mills M.J., Suter A., Lloyd A., Chavez-Montoya D., Civitelli D., Wolf B., Bolot P., Wasem J. (2025). FRAME: Framework for Real-World Evidence Assessment to Mitigate Evidence Uncertainties for Efficacy/Effectiveness—An Evaluation of Regulatory and Health Technology Assessment Decision Making. Clin. Pharmacol. Ther..

[21] Soriano A., Paterson D.L., Thalhammer F., Kluge S., Viale P., Watanabe A.H., Allen M., Akrich B., Wirbel S., Obi E.N. (2025). Unveiling results and insights from multinational, multicenter Study of Prescribing patterns and Effectiveness of Ceftolozane/Tazobactam Real-world Analysis (SPECTRA). J. Glob. Antimicrob. Resist..

[22] Yucel E., Soriano A., Paterson D.L., Thalhammer F., Kluge S., Viale P., Allen M., Akrich B., Levy J., Yang H. (2025). Real-world analysis of ceftolozane/tazobactam prescribing patterns and effectiveness: SPECTRA analysis on chronic pulmonary diseases and respiratory-related infections. J. Glob. Antimicrob. Resist..

[23] Soriano A., Paterson D.L., Thalhalmmer F., Kluge S., Viale P., Akrich B., Allen M., Wirbel S., Watanabe A.H., Yücel E. (2025). A real-world investigation into prescribing patterns and effectiveness of ceftolozane/tazobactam among critically ill patients from SPECTRA. Diagn. Microbiol. Infect. Dis..

[24] Kullar R., Wagenlehner F.M., Popejoy M.W., Long J., Yu B., Goldstein E.J.C. (2016). Does moderate renal impairment affect clinical outcomes in complicated intra-abdominal and complicated urinary tract infections? Analysis of two randomized controlled trials with ceftolozane/tazobactam. J. Antimicrob. Chemother..

[25] Lea-Henry T.N., Carland J.E., Stocker S.L., Sevastos J., Roberts D.M. (2018). Clinical Pharmacokinetics in Kidney Disease: Fundamental Principles. Clin. J. Am. Soc. Nephrol..

[26] Czock D., Keller F., Seidling H.M. (2012). Pharmacokinetic predictions for patients with renal impairment: Focus on peptides and protein drugs. Br. J. Clin. Pharmacol..

[27] FDA Labeling Information for ZERBAXA (Ceftolozane/Tazobactam). https://www.accessdata.fda.gov/drugsatfda_docs/label/2014/206829lbl.pdf.

[28] Li S., Fu Z., Zhang W., Liu H. (2024). Impact of obesity on intensive care unit outcomes in older patients with critical illness: A cohort study. PLoS ONE.

[29] International Society for Pharmacoepidemiology (ISPE) Guidelines for Good Pharmacoepidemiology Practices (GPP). https://www.pharmacoepi.org/resources/guidelines_08027.cfm.

[30] Word Medical Association Declaration of Helsinki. https://www.wma.net/.

[31] European Medicines Agency Guideline on Good Pharmacovigilance Practices (GVP): Module VI—Collection, Management and Submission of Reports of Suspected Adverse Reactions to Medicinal Products (Rev 2). https://www.ema.europa.eu/en/documents/regulatory-procedural-guideline/guideline-good-pharmacovigilance-practices-gvp-module-vi-collection-management-and-submission-reports-suspected-adverse-reactions-medicinal-products-rev-2_en.pdf.

[32] European Parliament and Council EU General Data Protection Regulation—Regulation (EU) 2016/679. https://data.europa.eu/eli/reg/2016/679/2016-05-04.

