# Supplementary material for: Prescribing Patterns and Clinical Outcomes of Ceftolozane/Tazobactam by Renal Function and Body Mass Index: A SPECTRA Real-World Multi-Country Analysis
Source: Antibiotics (Basel). 2026 Mar 17;15(3):303. doi: 10.3390/antibiotics15030303 (PMC13023874; doi:10.3390/antibiotics15030303)
Supplement: Supplementary file 1 [file antibiotics-15-00303-s001.zip › antibiotics-4113113-supplementary.pdf]

Supplementary material

Table S1. Renal replacement therapy types and duration*.

	End stage renal impairment	Severe renal impairment	Moderate renal impairment	Mild renal impairment	Normal renal function	Augmented renal function	Total study population
	N=22	N=48	N=127	N=153	N=229	N=18	N=617†
**New onset AKI during index hospitalization (%)**	14 (63.6)	23 (47.9)	56 (44.1)	30 (19.6)	30 (13.1)	4 (22.2)	161 (26.1)
**First RRT required during index hospitalization (%)**	9 (40.9)	11 (22.9)	21 (16.5)	10 (6.5)	14 (6.1)	2 (11.1)	71 (11.5)
**Type of RRT (%)**							
Intermittent hemodialysis or Peritoneal dialysis	3 (13.6)	6 (12.5)	7 (5.5)	3 (2.0)	3 (1.3)	0	24 (3.9)
Continuous renal replacement therapy	6 (27.3)	5 (10.4)	13 (10.2)	7 (4.6)	11 (4.8)	2 (11.1)	46 (7.5)
Extended daily dialysis / Sustained low efficacy dialysis	0	1 (2.1)	0	0	0	0	1 (0.2)
Other	0	0	1 (0.8)	0	0	0	1 (0.2)
**For patients with at least one RRT, total duration of the RRT (days)**							
Mean (SD)	30.0 (34.6)	45.2 (97.4)	15.9 (11.5)	20.4 (14.7)	24.8 (20.8)	21.0 (15.6)	25.1 (42.6)
Median	24	11	14	18	21	21	16
Q1;Q3	7.0 ; 27.0	7.0 ; 27.0	6.0 ; 21.0	11.0 ; 25.0	13.0 ; 29.0	10.0 ; 32.0	9.5 ; 26.5

*Creatinine clearance (CrCl) was derived and categorized per the Statistical Analysis Plan. Category cut points used for the table are: end stage <15 mL/min; severe 15–29 mL/min; moderate 30–59 mL/min; mild 60–89 mL/min; normal 90–149 mL/min; augmented ≥150 mL/min. Percentages in this table use the number of patients with non missing CrCl as the denominator (CrCl recorded in 597/617 patients). † The cohort size of the total study population was 617. Renal function was not recorded for 20 patients; percentages in this table use the 597 patients with non-missing CrCl as the denominator. RRT includes hemodialysis, continuous renal replacement therapy (CRRT), and other extracorporeal renal support modalities as recorded in site charts. 71 patients had RRT initiated during the index hospitalization. When a pre RRT serum creatinine suitable for Cockcroft–Gault estimation was available, the pre RRT CrCl was used to assign renal function category. Patients without an interpretable pre RRT creatinine were categorized as missing CrCl and therefore excluded from CrCl stratified denominators (CrCl recorded for 597/617 patients). AKI: Acute Kidney Injury; RRT: Renal Replacement Therapy; Q: Quartile; SD: Standard Deviation.

Table S2. Appropriate Dose of C/T for Index Event.

	CrCL level				
	**>50 ml/min**	**30–50 ml/min**	**15–29 ml/min**	**<15 ml/min**	**Missing**
**cIAI and cUTI**	1.5g/Q8h	750mg/Q8h	375mg/Q8h	375mg/Q8h then 150mg/Q8h	Not defined
**HAP and VAP**	3g/Q8h	1.5g/Q8h	750mg/Q8h	2.25g then 450g/Q8h	Not defined

cIAI: complicated intra-abdominal infections; CrCl: creatine clearance; cUTI: chronic urinary tract infection; HAP: Hospital-acquired pneumonia; Q8h: every 8 hours; VAP: Ventilator-associated pneumonia.
